# “An Unusual Pattern of Metastasis” Metastatic Malignant Thymoma Presented with Breast Lump: A Case Report and Literature Review

**DOI:** 10.1155/2023/3114843

**Published:** 2023-03-21

**Authors:** Kah-Seng Khoo, Kavinya Diana T. Nadesalingam, Diana Bee-Lan Ong, Li-Ying Teoh, Mei-Sze Teh, Suniza Jamaris, Mee-Hoong See

**Affiliations:** ^1^Department of Surgery, Faculty of Medicine, University of Malaya, Kuala Lumpur, Malaysia; ^2^Department of Pathology, Faculty of Medicine, University of Malaya, Kuala Lumpur, Malaysia; ^3^Breast Surgery Unit, Department of Surgery, Faculty of Medicine, University of Malaya, Kuala Lumpur, Malaysia

## Abstract

Metastatic lesions to the breast from extramammary malignant neoplasms are rare and reported account for 0.5–6.6% of all breast malignancies. Distant metastasis of thymoma is even rarer, especially to extrathoracic regions. We reported a woman with invasive malignant thymoma postneoadjuvant and resection of the thymoma, who developed breast metastasis 7 years later. Breast imaging showed high-density lesion with no intralesional microcalcifications and no significant axillary lymphadenopathy. Core biopsy and histopathology proved the lesion to be metastatic thymic carcinoma. Despite rarity, breast lumps with underlying extramammary malignancy should raise the suspicious of breast metastasis.

## 1. Introduction

Thymomas are neoplasms that arise from thymic epithelium. The World Health Organization classified thymomas based on the morphology of epithelial cells as well as the lymphocyte-to-epithelial cell ratio. They are broadly divided into 5 types (type A, AB, B1, B2, and B3) [1]. The staging of thymoma can be classified by Masaoka–Koga staging and divided into stage I until IV-B [1]. Thymic carcinomas are usually slow growing with local recurrence rather than metastasis. Majority of recurrences tend to be local. Metastases to the chest and into the breast are extremely rare. This care report is presented in accordance with Case Report guidelines [2].

### 1.1. Case Report

**Figure 1:**
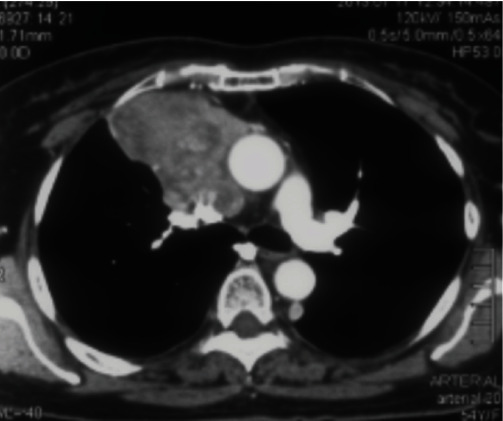
Huge thymic tumor with superior vena cava infiltration on CECT-TAP in 2013 (prior surgery).

A 61-year-old woman with a history of malignant thymoma was diagnosed in year 2013 with superior vena cava involvement (Masaoka–Koga staging III) (Figure 1). She underwent neoadjuvant chemotherapy (3 cycles of cisplatin and carboplatin) followed by oncological thymoma resection surgery. Intraoperatively, the thymoma was measuring 12 cm x 9 cm x 7 cm, adhered to the right upper and middle lobes of the lung, and it also involves the superior vena cava. En-bloc resection of the tumour with wedge resection of lung margins and interposition vein graft for superior vena cava was performed.

Final histopathology was reported as malignant thymoma. The tumour infiltrated the adjacent right lung and superior vena cava. The tumour showed positive involvement of the vascular resection margin and the anterior, inferior, and medial tumour resection margins. The lung resection margins were clear. A total of 8 lymph nodes retrieved were free of malignancy. Subsequently, adjuvant radiotherapy 60 Gy/30 fractions were given to the mediastinum. The patient was only able to tolerate 1 cycle of adjuvant chemotherapy (combinations of paclitaxel and carboplatin) due to the side effects.

**Figure 2:**
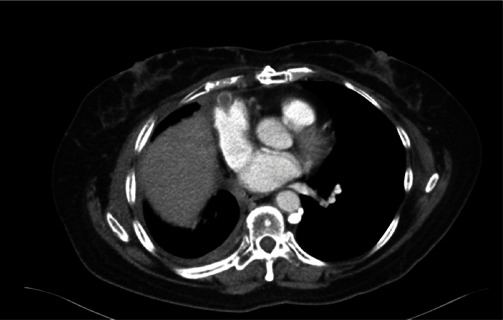
No evidence of breast lesion seen on CECT-TAP in 2016.

Three years after the surgery, surveillance imaging with contrast enhanced computed tomography (CECT) thorax showed increased lung nodules suspicious of metastasis in 2016 (Figure 2). The patient was offered palliative chemotherapy; however, she declined and wished for clinical follow-up only.

In January 2020, she developed dry cough and exertional breathless. Restaging imaging (CECT-brain, thorax-abdomen, and pelvic) showed progressing lung metastasis with incidental right breast lumps. There was no breast pain and nipple discharge. On examination, two breast lumps were palpable in the right breast. The first lump was measuring 5 cm x 5 cm and was mobile with no skin changes. The second lump was measuring 2 cm x 1 cm and was also mobile with no skin changes. Left breast was normal, and there were no palpable axillary lymph nodes on either side.

**Figure 3:**
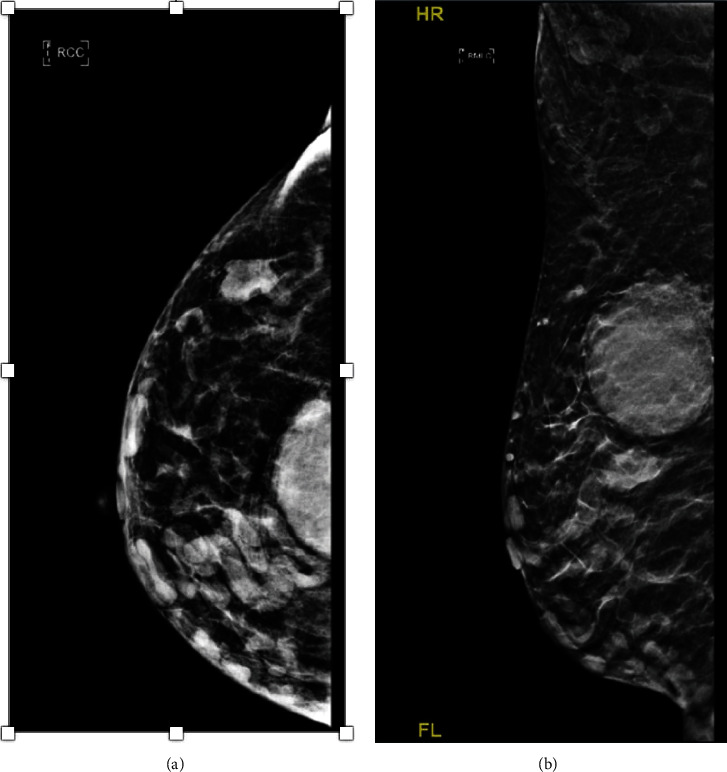
Mammogram of right breast showed well-circumscribed, round, high-density lesions at upper mid region and upper outer quadrants.

**Figure 4:**
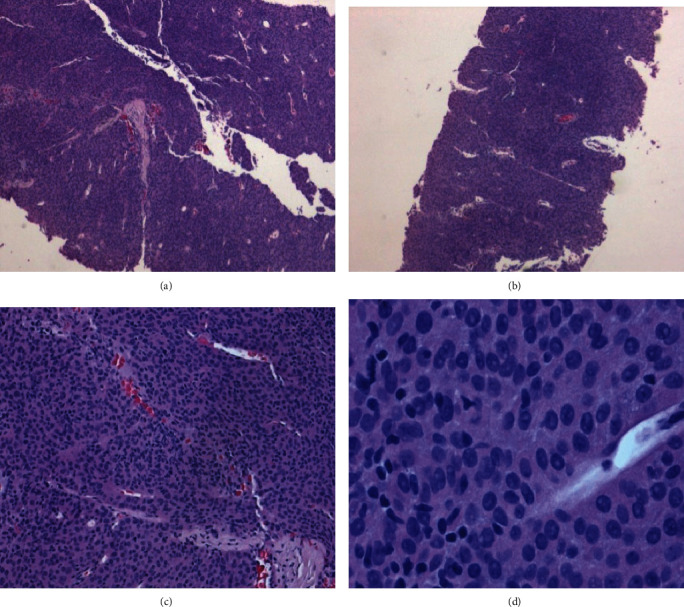
On histology, the lesional tissue is composed of (a) and (b) tumour tissue arranged in anastomosing smooth contoured islands, Hematoxylin & Eosin stain (H&E) ×40. (c) Absence of duct/glandular formation, H&E ×100. (d) Tumour cells have round to oval nuclei, inconspicuous nucleoli, and indistinct cell membrane, H&E ×400.

**Figure 5:**
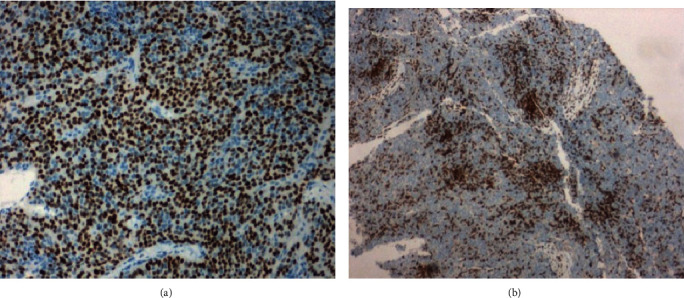
(a) Tumour cells show positive staining for p63 (×40). (b) CD5 shows patchy positive cells (×40).

Mammogram of bilateral breasts showed well-circumscribed, round, high-density lesions at upper mid region and upper outer quadrant of right breast (Figure 3). No significant microcalcification within. Complementary sonography showed Breast Imaging-Reporting and Data System 4b lesions, which were two well-defined heterogeneous lesions. The lesions correspond to the mammographic findings. Left breast was normal. There were no suspicious axillary lymph nodes on imaging. Tissue core-biopsy of right breast 12 o'clock lump was performed, and histopathology examination came back as metastatic thymic carcinoma (Figure 4), p53, and CD5 positive for staining (Figure 5). Histology was compared with the original thymic carcinoma. Multidisciplinary team discussion decided for palliative treatment and best supportive care due to her Eastern Cooperative Oncology Group 4 and disease progression with poor prognosis. She was given palliative care with home oxygen and analgesia. She came to oncology clinic follow-up until July 2020 and passed away in September 2020.

## 2. Discussion

Metastatic lesions to the breast from extramammary malignant neoplasms are rare and reportedly account for 0.5–6.6% of all breast malignancies [3, 4]. Thymoma is a tumour originating from the epithelial cells of the thymus, typically presenting in the 4th or 5th decade of life. Thymic carcinoma is rare with an incidence of 0.15 cases per 100,000 [1]. There are 3 ways thymic carcinomas can metastasize: hematogenous, or lymphatic routes, and direct invasion of adjacent organs [5]. Thymic carcinoma often metastasizes to the regional lymph nodes and variable extra-thoracic organs, particularly bone, lung, and liver [6]; 80% of cases have local invasion of contiguous mediastinal structures, and 40% of cases present with metastatic spread to bones, lung, pleura, liver, or lymph node [7].

Differentiating between primary breast malignancy and metastatic deposit will be extremely difficult clinically, because the presentation and examination will be quite similar between the two spectrums of disease. However, patient's personal history of underlying malignancy could raise the suspicion of metastatic deposits as differential diagnosis. Metastatic lesions may simulate primary breast carcinoma or benign abnormalities radiographically. Hence, accurate diagnosis will require appropriate histopathology and immunohistochemical examinations, along with review of histology of any previous cancer for comparison.

Reports of metastatic breast tumours from the thymus are rare. To the best of our knowledge, there are 4 documented case reports of thymic carcinoma with metastasis to the breast. The first published report documented the findings of distant metastasis to the breast, after the 5th cycle adjuvant chemotherapy for a resected poorly differentiated thymic carcinoma [8]. A second case report described a woman with initial non-invasive thymoma that developed metastases 20 years after a thymectomy [9]. This signifies that thymic tumour have the potential to metastasize despite long duration of disease free period. Another case report noted that a woman with underlying type AB thymoma developed breast metastasis 10 years after complete tumour resection [10]. The most recent case report described a woman with poorly differentiated squamous cell carcinoma of the thymus that developed breast metastasis 3 years after completion of chemo and radiotherapies [11].

When dealing with thymic carcinoma, it is critical to determine whether the mass can be surgically resected and total thymectomy with complete surgical excision of tumour is recommended when possible. Patients with completely resected tumour have longer survival than those who are either incompletely resected or unresectable. R0 resection has a 5-year survival of about 60%. After surgical resection, post-operative management includes radiotherapy with (or without) chemotherapy, depending on the completeness of resection. For our patient, she had R1 resection, as evidence by the final histopathology report showed positive involvement of the vascular resection margin and the anterior, inferior, and medial tumour resection margins. Hence, she was offered adjuvant chemo-radiotherapy. Unfortunately, thymic carcinomas respond poorly to chemotherapy, and first-line therapy is carboplatin/paclitaxel (overall response rate, 22–36%) [12]. Generally, the treatment of metastatic thymic carcinoma would be palliative systemic therapy, which include chemotherapy, and some evidences proven targeted therapy effective.

Although there are no clinical trials providing evidence of benefit, monitoring for recurrences with thoracic imaging on an annual basis is warranted. For patients with recurrent disease detected on surveillance, early intervention may be more feasible and effective [13]. Surveillance for subsequent primary cancers is also indicated, as higher incidence of other tumour types has been reported in patients with thymomas. The guidelines from National Comprehensive Caner Network recommend commuted tomography every six months for 2 years, then annually for five years for thymic carcinoma [13]. Recurrence of thymoma may not become apparent for many years after initial treatment. As an example, in a series 126 patients who underwent complete resection of a thymoma, 24 eventually recurred [14]. The time to recurrence ranged from 4 to 175 months (mean, 68 months). The initial sites of recurrence were pleural, local, and distant in 22, 6, and 5 cases, respectively.

In conclusion, despite the rarity of extramammary breast metastasis, a patient presenting with breast lump with an underlying history of thymic carcinoma, even long time after initial presentation and treatment, should be worked up for a possible metastatic disease.

## Data Availability

Data will be stored in the department computer.
